# Regulation of growth and metabolism

**DOI:** 10.1186/2049-3002-2-S1-O20

**Published:** 2014-05-28

**Authors:** David M Sabatini

**Affiliations:** 1 Whitehead Institute for Biomedical Research; Howard Hughes Medical Institute and Department of Biology, MIT; Broad Institute of Harvard and MIT; The David H Koch Institute for Integrative Cancer Research at MIT, Cambridge, MA, USA

## 

mTOR is the target of the immunosuppressive drug rapamycin and the central component of a nutrient- and hormone-sensitive signaling pathway that regulates cell growth and proliferation. We now appreciate that this pathway becomes deregulated in many human cancers and has an important role in the control of metabolism and aging. We have identified two distinct mTOR-containing proteins complexes, one of which regulates growth through S6K and another that regulates cell survival through Akt. These complexes, mTORC1 and mTORC2, define both rapamycin-sensitive and insensitive branches of the mTOR pathway. I will discuss new results from our lab on the regulation and functions of the mTORC1 and mTORC2 pathways.

